# Health and Masculinities Shaped by Agency within Structures among Young Unemployed Men in a Northern Swedish Context

**DOI:** 10.1371/journal.pone.0124785

**Published:** 2015-05-08

**Authors:** Anne Hammarström, Berit Lundman, Christina Ahlgren, Maria Wiklund

**Affiliations:** 1 Dept. Public Health and Clinical Medicine, Unit of Social Medicine, Umeå University, Umeå, Sweden; 2 Dept. Nursing, Umeå University, Umeå, Sweden; 3 Dept. Community Medicine and Rehabilitation, Physiotherapy, Umeå University, Umeå, Sweden; UNC School of Dentistry, University of North Carolina-Chapel Hill, UNITED STATES

## Abstract

**Aim:**

The aim of our paper was to explore expressions of life choices and life chances (aspects of agency within structures) related to power and experiences of health among early unemployed adolescent young men during the transition period to adulthood. These expressions of agency within structure were interpreted in the light of Cockerham’s Health Lifestyles Theory. Furthermore, social constructions of masculinities were addressed in our analysis.

**Methods:**

Repeated interviews with ten young men in a cohort of school leavers were analyzed with qualitative content analysis.

**Results and Discussion:**

Cockerham’s model was useful for interpreting our findings and we found disposition to act to be a crucial theoretical tool to capture the will and intentions of participants in relation to health. We developed the model in the following ways: structure and socialization were visualized as surrounding the whole model. Analyses of what enhances or restricts power are important. In addition to practices of health lifestyles, we added experiences of health as outcome as well as emotional aspects in disposition to act. We interpret our findings as constructions of masculinities within certain structures, in relation to choices, habitus and practices.

**Conclusions:**

Qualitative research could contribute to develop the understanding of the agency within structure relationships. Future studies need to pay attention to experiences of health among young people at the margin of the labor market in various milieus – and to analyze these in relation to gender constructions and within the frame-work of agency within structure.

## Introduction

The increasing rate of youth unemployment has become a major global problem. The stress of constructing masculinity amongst the unemployed may be related to the stigma of not being able to construct a social identity through work and breadwinning. In a study of working-class men on the margin of the labor market, Connell ([1], p.164) concludes that they construct gender from ‘a starting point in poverty, and with little access to cultural or economic resources’. Unemployment among youth increases the risk of both mental illness and poor health behavior [2]. However, there is a lack of comprehensive theoretical models in order to understand the development of poor health among the unemployed [3]. There is a need to develop broad and comprehensive theories within the field [3]. Here, the framework of agency within structure could be valuable. By definition the unemployed are limited by structures in the sense that they lack employment possibilities. Thus, in this respect the structures generate powerlessness over their working life. Power relations are central in *structures*, which could be defined as sets of ‘resources that empower or constrain social action and that tend to be reproduced by that social action’ ([4], p.19). In health promotion, power and empowerment can be linked to high self-esteem and positive health outcomes, whereas powerlessness is typically connected to adverse health experiences [5–6]. Power, wealth and self-esteem are then seen as tied to an individual’s position in society or work-place [6]. *Agency* refers to how individuals act within (or despite) social settings [7]. Thus, individuals can use agency to negotiate, challenge and transform their contexts and the surrounding intersecting power-structures, related to for example gender and social class. However, the agency within structure model is underdeveloped in medical sociology [8] as well as within public health [9]. In addition, gendered dimensions such as constructions of masculinities in relation to experiences of health need to be developed within the model.

Medical sociologists, such as Williams [10–12] and Cockerham [8,13] have emphasized the lack of frameworks which link agency within structure models to experiences of health and health behavior. As a response, Cockerham [8] has developed a Health Lifestyle Theory in which he defines health lifestyles as ‘collective patterns of health-related behavior based on choices from options available to people according to their life chances’ ([13], p.249–250). Structural variables such as social class and gender, collectivities (which he defines as ‘collections of actors linked together through particular social relationships, such as kinship, work, religion, and politics’ ([8], p.59) and living conditions provide the social context for socialization and experience which influence life choices (agency). From this view, primary socialization represents ‘the imposition of society’s norms and values by significant others’, and the secondary socialization results from later and ‘learned outcome of day-to-day activities that comes about through social interaction and the practical exercise of agency’ ([8], p.60). For young people, significant others mainly comprise parents, siblings, peers and partners. Schools, community youth centers, workplaces and the close neighborhood are some of the social contexts which potentially influence norms and values.

The structural variables also constitute life chances (structure). Life choices and life chances interact in the formation of dispositions to act which Cockerham defines based on Bourdieu’s concept of habitus [14]. He defines ‘disposition to act’ as reflecting an individual’s habitus which builds on former experiences and socialization, as well as on class circumstances and other social categories (e.g. gender, age, ethnicity), collectivities and living conditions [8]. Habitus then serves as ‘cognitive maps’ or a ‘set of perceptions’ that routinely guides choices and actions in particular social settings and situations [8]. Disposition to act leads to practices, which constitute patterns of health lifestyles and in turn feedback to the habitus.

Cockerham [8] suggests that gender is ‘a significant variable’ for healthy lifestyles. A gendered analysis applies his approach in combination with Bourdieu’s theory of lifestyle to body weight among Danish women [15]. In order to further develop gender in his model, social constructions of masculinity can be seen as a crucial part of young men’s socialization processes, situated in a certain social context and time. In the context of Northern Sweden, Sandström and Dahlgren [16] have formulated a set of ‘ideal-types’ of adult masculinities that were present in the region in the 1980s:‘the real man’; ‘the working class man’; ‘the independent man’; and ‘the anomic man’. Thanks to the similarities in time and context these empirically grounded, but theoretically constructed, adult masculinities may also be relevant for the young men in our study. These masculinities convey different attitudes to work and health ranging from a traditional construction of collectively oriented masculinities with a strong focus on hard work and solidarity with work-mates and the employer, including working despite pain, to more independent and individualistically-oriented masculinities. Such masculinities can be understood as being subject to change in relation to the wider society, for instance to the transitions of the Swedish welfare state and the still today powerful positions of male-dominated trade unions.

Thus, in the context of Northern Sweden during the 1980s, the dominant and normative way of constructing masculinity and social identity was through hard physical work. Consequently, long-term unemployment in early ages challenged the normative pattern. However, in several respects this time-period can be defined as a historical and political break-point in the Swedish society, both in terms of early signs of emerging changes in the welfare state ideals towards more individualistic neoliberal ideologies and policies, but also in terms of changing gendered patterns at the labor market and in domestic arenas. The main driving forces here were women’s liberation and strive for equal rights with women entering the labor market to a much larger extent than before.

Cockerham [8] concludes that his theory needs verification, change or rejection through empirical application. In later publications [13], he argued for the need of not only quantitative but also more qualitative empirical studies. The only qualitative empirical application of his model which we have found is a study among rural adults about symptoms leading to physician consultation [17]. These authors find the Cockerham model useful due to its focus on both general dispositions and symptom-specific responses. However there is a lack of other empirical studies which have tested or used Cockerham’s Health Lifestyle Theory empirically.

According to Cockerham, qualitative research using the agency within structure framework has considered agency, but few studies have also addressed the structural influences.

Therefore, the aim of our study was to explore expressions of life choices and life chances (aspects of agency within structures) related to power and experiences of health among early unemployed adolescent young men during the transition period to adulthood. These expressions of agency within structure were interpreted in the light of Cockerham’s Health Lifestyles Theory. Furthermore, social constructions of masculinities were addressed in our analysis.

## Methods

### Setting

The cohort study, from which the interviews for this analysis were drawn, was performed in a middle-sized (with about 70 000 inhabitants) industrial residential town in Northern Sweden during the 1980ies.The town has been shown to be representative for Sweden as a whole in relation to socio-demographic profile, health status and health habits among young people as well as the labor market conditions except for an endemic high unemployment rate which during the 1980s was twice as high in Northern Sweden than in the country as a whole [18].

The town has an important harbor and the labor market is dominated by a steel company, a technical university college, the Swedish Armed Forces as well as by the public sector, especially the County Council. During the 1980s it was characterized by higher workforce immigration from Finland than the rest of Sweden. In political terms, the area had for a long time been dominated by the Social Democrats together with the smaller socialist parties, indicating that the northern part of Sweden was the most left-wing orientated in the country. In addition, religious activities were quite low.

In order to tackle the growing rate of youth unemployment, the Swedish government made large investments in active labor market policy measures directed towards young people from the beginning of the 1980s. These labor market policy measures provided educational and vocational activities for unemployed, while payment was provided by the state. The most common labor market measure for young people in the early 1980ies was subsidized employment, with work tasks that did not compete with regular jobs on the labor market. The objective of the labor market policies was that no one should be unemployed in the age group 16–18; either they should be studying at high school, working or participating in labor market measures. In 1980, active labor market policy programmes for young people were introduced which were intended to guarantee eight hours daily work time during 40 weeks, paid with study assistance rather than with a salary. In 1982, the time for reimbursement for these programs was somewhat increased beyond 40 weeks. In 1984, a law for 18–19 year olds was introduced expressing that no one should be unemployed but instead should be participating for at least four hours per day in labor market measures to a minimum salary [19]. This meant that in international comparison, Sweden had a very active labor market policy towards young people during the 1980s. For the participants in our study, these active labor market policy programmes meant that they did not become permanent long-term unemployed but rather moved between unemployment, various labor market programs and shorter periods of temporary employment.

### Participants, design and data collection

The Northern Swedish Cohort—a prospective longitudinal cohort study—consists of all pupils (n = 1083) who in 1981 were in their last year of compulsory school (age 16) in nine schools in a middle-sized town in Northern Sweden. The cohort has been regularly followed over time by the PI (AH) with questionnaires about health status and life circumstances [18].

A subsample of this cohort consists of all young men (n = 10) who became unemployed directly after compulsory school. They all had working class backgrounds and many of them had so much school truancy that their grades were not high enough to continue to secondary high school.

This subsample of early unemployed men has been followed with personal interviews by the PI since autumn 1981. Most interviews were conducted between age 16 and 22 and therefore these have been chosen for analysis in this paper. During this period the men were interviewed between two and three times per person. In total, 22 interviews were conducted with the ten men from age 16 until age 22.

Individual audio-recorded structured interviews were performed by the PI, a medical doctor and specialist in social medicine. The interviews reflect her interest in social and material life circumstances as well as the possibilities of controlling one’s life. The questions dealt with daily activities in various spheres, possibilities of influencing one’s life, dreams for the future, and experiences of health and unemployment/work. Many of the young men were quietly spoken and did not say much in interviews, which is a typical trait of men in this region. Consequently, although the interviews were quite informative, they were not very rich in experiences, feelings and thoughts.

### Data analysis

The interviews were analyzed by using qualitative content analysis as described by Graneheim and Lundman [20]. Content analysis is a method of analyzing written and verbal communication in a systematic way [21]. The approach can be both data-driven and theory-driven [22]. In this study we have used a combination of data-driven and theory-driven analysis.

The interviews were read through several times by all authors to get a sense of the whole. Thereafter, the analysis was performed in several steps. First, the text was divided into meaning units, each comprising several words, sentences, or paragraphs containing aspects related to each other through their content and context. After that the meaning units were condensed, i.e. by shortening the text while still preserving the core. The condensed meaning units were labelled with a code and sorted into content areas. A content area is a rough structure of content that can be identified with little interpretation. The content areas were about work, school, unemployment, action plans, the family, daily activities, leisure activities, economy, future, way of living, health, wellbeing, experiences of control and possibilities of influencing the circumstances of one’s life. The analysis was repeatedly discussed in the research team to strengthen trustworthiness.

A red thread of meaning through all of the content areas was identified as experiences of power (or agency) and powerlessness (lack of agency) which led to a focus on what enhanced or restricted power. Examples of initial categories for enhanced power were: the importance of having paid work, the benefits of unemployment, active spare time, hopes for the future, good health, supportive relations with parents, girlfriends and mates. Examples of initial categories for restricted power were: difficulties of finding work, loss of daily routines, uncertainty about the future, ill health, and strained social relations.

To be able to contextualize the findings and simultaneously address issues of agency and power, we found it more meaningful to frame our analysis by a theory than to present our results as categories. We found it suitable to use the model of agency within structure in connection to health experiences and health behavior. We also believed this was an advantage as our data was collected in a historical and political break-point and as the participants were not so talkative. Therefore, we turned to the literature and found it suitable to use the Health Lifestyles Theory, developed by Cockerham [8] to interpret our findings. This was the theory- driven part of our analysis.

### Ethics statement

Ethical approval was during the study period granted by the ethics committee of Uppsala University and Umeå University as well as by the Regional Ethical Review Board in Umeå. According to the Swedish law (Swedish Ethical Review Act 2003;460, § 17), there is no requirements of written consent of the participants of an interview or questionnaire study. In addition, written informed consent was not requested by any of the ethic committees, as the participants are regarded as giving written consent when agreeing to participate in an interview or completing a questionnaire. All participants were clearly informed that participation in the study was voluntary and that they could decide to withdraw from participation at any time, without giving any explanation.

The parents of the minors enrolled in the study were informed about the study. Written informed parental consent was not requested by the ethic committees.

The ethics committees approved our whole consent procedure.

## Results

Our findings are based on our analysis of the ten young men’s narrated experiences of health in the context of economical marginalization and early unemployment after leaving compulsory school. Their living conditions and socialization were characterized by their working-class belonging. In one sense this belonging was related to dignity and proudness because the area had a long tradition of powerful and masculine dominated working-class trade unions. But in comparison to the other young men in the cohort, these ten men had the worst material and psychosocial living conditions in terms of poor relations to others and lack of influence over central aspects of their lives as well as the poorest school experiences [19].

As part of their shared collectivity and socialization, the young men articulated an anti-school culture since a majority had had bad experiences in school. In addition, some described themselves and their peers as often being part of quarrels and conflicts, or even being violent, expressed as ‘when you do not have words you fight…I could with a wink rule the whole town’. An alcohol- and drug-culture was also present as seen in the following interview excerpt:

*Researcher (R)*: *You are unemployed now*, *what happened*?

*Participant (P)*: Yes, I quit [a labor market measure] as planned after six months. Then they should have prolonged my contract but then I ended up in a damned quarrel with them and therefore I quit.

*R*: *Why was there a quarrel*?

*P*: It was the youth center that started to nag on me and asked if I was using hashish and all other possible kind of things

*R*: *Why did they think you used hashish*?

*P*: I came one day, and they thought I began to become spaced-out

*R*: *OK*, *then you started to quarrel and what happened then with the job*?

*P*: Yes, we started to quarrel. Yes, they suspended me.

*R*: *Did they*? *They did not believe you*?

*P*: No.

*R*: *So they did not trust you*, *was that what the quarrel was about*?

*P*: Yes, but if I shall be honest I was a bit lazy so to say, I was very tired, getting up early in the morning every day…

**Fig 1:**
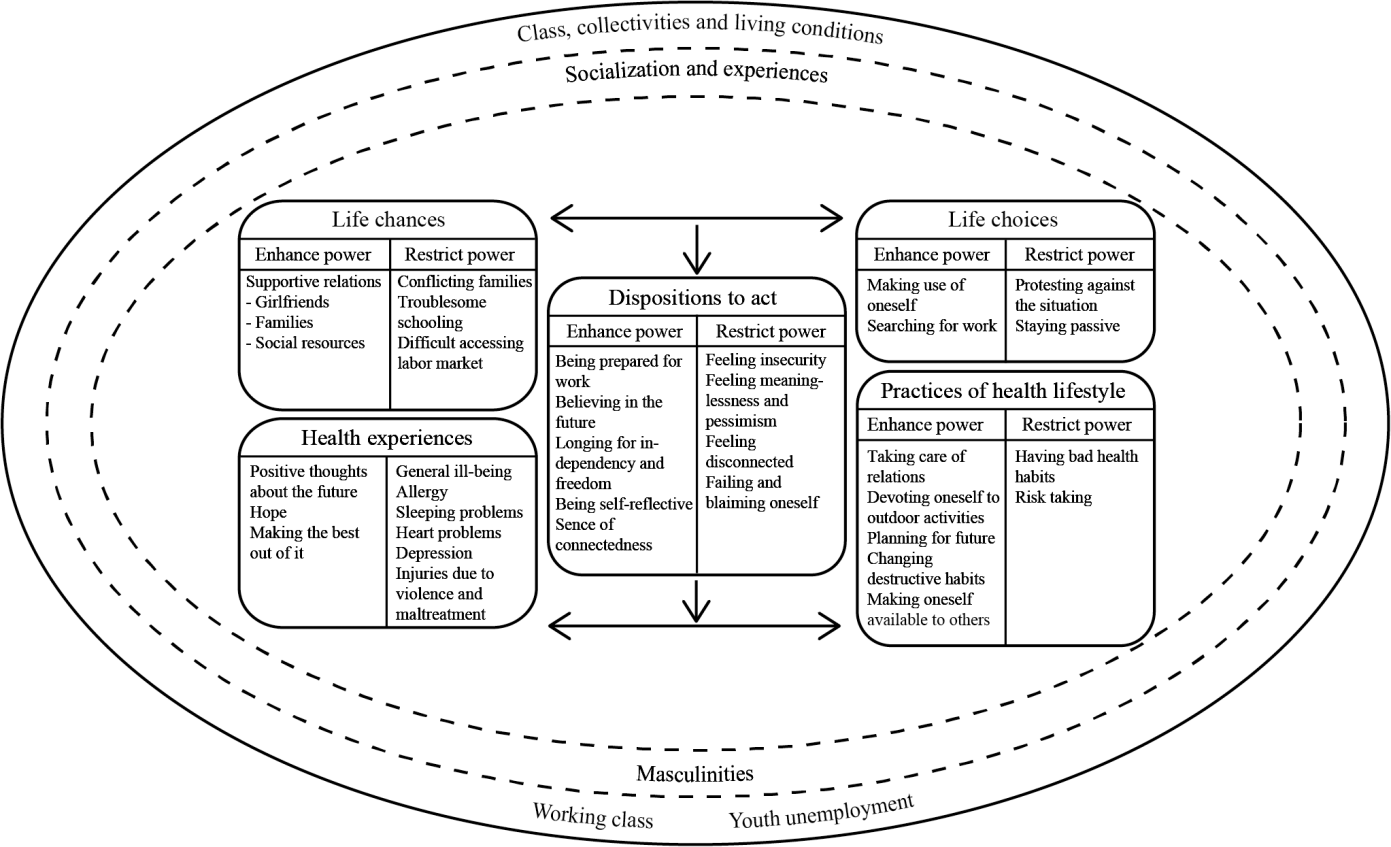
Model of life choices and life chances related to power and experiences of health. Our figure is a development of Cockerham’s health lifestyles theory within the framework of agency within structure [8].

The interview excerpt illustrates the behavior of a young man along with his experiences of being accused and distrusted by people in his close surroundings such as professionals at the youth center, his employer and his physician. It reflects the day-to-day social interaction between the individual’s actions and the social structures/environments, which were part of the socialization and experiences of the young men. All these processes framed the shared social context for understanding the results (see also the outer circles of Fig 1 below).

### Life chances (structure)

The young men’s structuring conditions, here termed as life chances, were constituted by their working class circumstances, poor living conditions, and masculine collectivities, as well as by their young age.

Life chances represent structures which enhance or restrict power, and were related to advantages and disadvantages of relative class situation. We have divided our results regarding life chances into chances which enhance power and ability to act as a contrast those which restrict power.

#### Life chances which enhance power

In our interviews, life chances which enhance power were strongly related to supportive social relations, especially with girlfriends but also with parents, and, to a lesser extent with friends, as well as with societal institutions such as school.

Supportive girlfriends were described as having a strong influence in several different ways. Having a girlfriend was depicted as important support for calming down after having lived a risk-taking life in relation to alcohol. Before having a girlfriend you could show off by being out and ‘carry on during the weekends’ (see also ‘practices of health lifestyle’ below). Thus, finding a girlfriend was related to diminished alcohol consumption as well as a calmer life. But a girlfriend was also described as important because you could talk to her; she could give hope to your life and could make you feel less restless.

Supportive relations within the family could also be related to the young men’s ability to act. At age 21, normally most young people would live by themselves, but in the interview group some were still living at home. For example, one man said that he liked to live with his parents as long as he could manage to do that without fighting and arguing with them. He meant that he needed the various kinds of support offered by his parents; otherwise he would lose control over his life and be out drinking and living a dangerous life. Unemployment meant lack of income and the fact that some parents accepted that their sons lived at home meant that they could survive without an income. Parental support could also mean increased power by providing the sons with a job or helping them to find a job.

The young men’s ability to act increased when they got support from societal institutions such as school, workplaces or organizers of active labor market policy programmes for youth. One unemployed young man, who had become depressed due to a most problematic family situation, felt ‘perfect’ when he got a youth unemployment measure at a garage where he mended cars. He described what the work meant to him:

Yes, to have good contact with the owner as well as with the others. And to come to a place where you feel you can perform something.

#### Life chances which restrict power

Life chances which restrict support and thereby power and ability to act were related to conflicting family relations, troublesome schooling as well as to difficulties accessing the labor market.

The family relations could not only be supportive but also problematic in several ways. The young men told us emotionally strong stories about poor relationships with their parents. Mothers could be experienced as nagging or fretful. Due to parental separation or divorce fathers were often experienced as absent and the young men lost contact with them. The mother of one man had alcohol problems while another had a new partner who was experienced as very hard-hearted. Problematic relations within the new step-father led to this man fleeing the home at an age of only 16 Despite support from the social welfare service to find an apartment, his loss of family relationship made him dependent on older young men with risky health behavior (including misuse of alcohol) and he himself also increased his alcohol consumption which in turn led to increased passivity and decreased possibilities to act.

Troublesome schooling was another problematic life chance which could lead to interrupted studies and thus fewer future possibilities to find employment on the labor market. Strong stories were told about problematic experiences at school, expressed in terms like: ‘I hated every day at school’. Many of the problems seem to be related to teachers who could be experienced as unfair or unwilling to understand their efforts. The interviewees’ could be aware of the need for high grades in order to be able to continue their studies but despite own ambitions to raise their grades, the teachers lowered them. The young men talked about conflicts with the headmaster and with teachers. One man described how he had changed school in the last year of compulsory schooling and how he then really tried to improve his grades:

But the teachers thought that I talked nonsense. Most of them started to hate me. And of course I hated back.

Thus, a negative spiral was described of lack of support and negative response from important social institutions and structures, such as teachers in school, leading to decreased possibilities to make active choices (in this case in relation to further education) were described.

Difficulties in accessing the labor market were other structures (life chances) which restricted the possibilities to get a job and thus the power to influence your life. Men with long-term experiences of unemployment felt they did not get enough support from the Employment Office to find a new employment which made them feel insecure about their future in relation to job opportunities. Young people without further education were marginalized on the labor market and had to accept any available job, independent on work environment and work tasks. Thus, when the young men got a job or a labor market measure the actual work tasks could be experienced as boring or stressful. In addition, relations with the employer were not always easy. Thus, one man narrated that he seldom got credit from his boss although the other employees appreciated him. Thus, the boss did not prolong his employment contract and he became unemployed again.

#### Life choices (agency)

In our data life choices or agency were closely linked to the young men’s handling of their life situation as unemployed in a working class community. Not having a job meant that you had too much spare time which was perceived as boring and also made them economically dependent on their parents. The situation threatened their self-identity as ‘a real man’. In their behavior to uphold this constructed masculinity, some of them chose to adapt to working class ideals, and take on all work opportunities, while others protested against them and choose a more laid back behavior. We interpreted the first behavior as enhancing their power and ability to act in the eyes of significant others, and the laid back behavior as restricting their ability to act.

#### Choices which enhance power

The young men in our study constructed gender in line with the ideal characteristics of a hard-working masculinity. They constructed themselves as respectable citizens, who contributed to the community with their work as young men who were independent and made their own living. Physical strength was important in their masculinity construction both as a man, who never hesitated to take on hard work tasks, and a man upon whom less strong persons (mothers, elderly and sick or disabled neighbors) could rely on help with difficult tasks.

The young men’s striving to find employment was a key focus. By taking on all job opportunities offered and regularly going to the Employment Office, they retained their image of being a real man, despite times of unemployment. In this way the men constructed a normative masculinity, which also enhanced their possibilities to find a job and thus their ability to act. The cause for unemployment could not be laid on someone who took on all job opportunities that came their way. Another strategy for improving their life chances was through net-working, as they approached employers and asked for jobs. They were also prepared to move to the South of Sweden for finding a job as illustrated by a young man who had temporary work as a lorry driver:

They [a carnival] were here in [the town] and I went and asked if they had any temporary [employment]…Yes, I was there over the weekend and packed and packed and the boss came and asked if I wanted to follow them, they needed a lorry driver… so it happened that I could do what I wanted most of all; to drive a lorry.

To make one-self available for all sorts of work tasks was illustrated by a man working in a printer agency. He took on all sorts of work tasks, which resulted in him being offered full-time employment with a certain degree of freedom to organize his own work:

Overall, I come and go as I want to. I have six or seven permanent tasks which I need to do every day. As long as I perform them properly, they [the employers] are happy and after that I am quite free to take on other tasks.

‘Making use of oneself’ reveals the interviewees’ struggle to build an identity as a worthy citizen despite being unemployed. Unemployment was a threat to their construction of a real man and they took on all kinds of ‘job-like’ activities to gain respect in the community. They really wanted to work, helped family members or neighbors with work tasks, often without economic payment. Offering help to others with physically hard work tasks enhanced their pictures of being masculine further both in the eyes of others and themselves. This helped them to maintain their self-respect. Inactivity was perceived as stressful and ‘unmanly’.

This was illustrated by one of the participants, when he answered a question on which work he liked best. At the time he worked thirteen hours a day seven days a week as a lorry driver for a man in the village and also worked in his father’s forest. He related: ‘both of them are equal fun, but most of all I like hard physical work. It is nice to work, it feels good’.

#### Choices which restrict power

‘Protesting against the situation’ and ‘staying passive’ were identified as choices which restricted the ability to find an employment among certain young men. These men constructed a more disconnected and anti-social masculinity. The most negative aspect of unemployment in their eyes was a shortage of money and that their days became boring. Protests against the situation were expressed as active actions and were primarily exercised as moving in and out of education or leaving temporary employments. The protests had often started as truancy from school and as a wish to avoid demands in favor of they described as ‘…to have fun instead’. A young man, who had left a temporary job, because the employer wanted him to work harder and be there on time said:

I think I worked enough for the low payment …… well, to be honest, I was a bit lazy, or too tired. You had to get up at seven o’clock every morning, or you actually had to be at work at seven.

‘Staying passive’ describes unemployment as a passive situation and was expressed by those interviewees who made no active plans but instead took it easy, eventually moved around in the village and followed others in their activities as they came up. Periods of unemployment were experienced as boring. But they could not mobilize the energy required to find a new job. They postponed important contacts with Employment Office staff or just let contact with them ebb away as described by this young man;

Yes, you slept during the days, went out during the nights and it is very easy that you end up in those things.

Although such behaviors could give positive response from other young men in their close environment, the employment officers and employers would get a negative impression of their will to work which diminished their chances of finding a new job.

#### Dispositions to act (habitus)

We interpret that participants’ different dispositions to act reflected their habitus, which was built on their classed and gendered experiences and socialization. Thus, their habitus, here expressed in perceptions and cognitive maps, guided their daily choices and actions—as well as their thoughts about the future.

#### Dispositions to act which enhance power

Dispositions to act interpreted as enabling power comprised being prepared for work, believing in the future, longing for independency and freedom, being self-reflective and sense of connectedness.

Being prepared for work concerned some of the young men’s positive thoughts about and attitudes to work, which related to their expectations on becoming working (class) men. The young men differed regarding how they viewed and talked about work: either as something positive and meaningful, or as a ‘must’ and burden. Among those who talked about work as something they were prepared for, strived for, and viewed as meaningful, expressions like ‘I want a job’, ‘Having a job is important’, ‘I take any job’, ‘I can take all jobs except cleaning’, or, ‘If you do something meaningful during the days one can do other things in the evening’ were put forward. For many, the ultimate and ideal situation was to have a job and a stable income in the future, which also was connected to the ability to fulfil dreams.

The cognitive map of believing in the future was interlinked with having dreams and hopes, as well as with the vital sense of actually having a future. Dreams and hopes were related to work, housing and travels as well as to family. The dreams about work either concerned the actual type of work, for instance being a tractor or long-distance lorry driver, or the form of work, for example dreaming of running a small business of one’s own.

R: What about you, what will you do in ten years time?

*P*: *Yes*, *I will work and have a car*, *an apartment*

R: What do you think you will work with or what would you like to work with?

*P*: *I do not know—I would most of all like to work with something mechanical*

When dreaming of housing they talked about buying a house instead of living in a flat. One participant told of his dream of rebuilding his parents’ house, whereas others wanted to move from their parents. Their thoughts about the future were often connected to their longing for independency, mobility and freedom, which also worked as a driving force for trying to get a job and creating a respectable social identity, by some expressed as ‘money gives freedom’.

I would like to have a job in order not to have to live off my father.

The young men differed regarding how much they reflected about themselves and their current or future situation. Some had realistic and ‘down to earth’ plans, whilst others had somewhat unrealistic dreams, far from their current situation. Several tried to make the best of their situation—although some also marked distance by noting that their dreams were mostly ‘empty talk’. One participant reflected on his present work and life situation and hoped for the best, put as ‘one has to take it with afterthought, one cannot say that it becomes super’. Another participant concluded that he worked because he ‘had to work’, implying that he had lowered his expectations of getting meaningful work. These examples show how some of the young men adapted their goals and plans to their assumed restricted possibilities (constrained by structures), and in some ways enhanced their power to act by accepting or adapting to the given situation.

Sense of connectedness was another disposition which we interpret as enabling the participants’ social identities and power to act (despite restricted possibilities on the labor market) in line with present social and masculine ideals. This sense of being connected was also related to aspects of ‘making use of oneself’ by working and helping others and to ‘being prepared for work’, as described earlier. It illustrates a caring and connected masculinity tied to a feeling of being part of and belonging to the community, and as linked to one’s own actions and contributions to the social day-to-day interactions of ‘give and take’ in the community. This aspect of the agency-structure relationship relates to a feeling of being useful and having a social value (to be compared to the opposite disposition and social position of feeling disconnected).

#### Dispositions to act which restrict power

Dispositions to act which constrained power comprised feeling insecurity, feeling meaninglessness and pessimism, failing and blaming oneself and feeling disconnected.

Feeling insecurity in relation to their present and future situation was linked to indifference and powerlessness. The young men had scarce or no hope of finding a job, and had difficulty imagining or planning for a future:

R: How do you think it will go then, will you get a job?

*P*: *I don’t know*, *I hardly think that*.

R: What do you think you will do in ten years?

*P*: *No*, *I don’t know*

R: When you are 25 years old?

*P*: *I don’t know*.

Thus, *feelings of meaninglessness and pessimism* were related to lost hope of finding a job and a general lack of life-goals. In this context, mistrust of professionals and ‘the system’ was also expressed. For instance, the young man below, who described himself as lazy and indifferent, saw it as meaningless to go to the employment office:

First of all, I am too lazy. It takes such a long time before I get the unemployment benefit. You must work half a year to the benefits. Then I believe that it is meaningless. Total meaningless … I have not been there [Employment Office].and will not put my foot there

He described how he had gradually started to view things from a more pessimistic perspective, and thought it would ‘go to hell’, now when he realized what life would be like:

I become more depressed as the years go by. Yes, everyday one sees more of the reality. I am trying to fight it well, I am trying to survive it, but I hope I never get older. I will never become a day over 16 years, that’s my highest hope. I’ll never get old.

Failing and blaming one-self was another disposition to act which reflected the young men’s restricted power and agency connected to low self-esteem and scarce belief in themselves and their abilities. This also included their view of themselves as having failed in life, as well as their low influence on their lives. Setbacks were commonly understood as ‘it’s my own fault’. In this context of pessimism and meaninglessness, a masculine disposition of opposition and not wanting to live up to mainstream societal norms and responsibilities was formulated as *being disconnected*. This was closely linked to the choice of ‘protesting against the situation’, as earlier described. Some rebuffed ‘respectable’ life choices in line with normative working class values of becoming an ordinary ‘Joe’. Instead, they wanted to be free from responsibilities and go their own way in life. For one, the age when you are permitted to drive a moped bike was still the happiest time in his life.

### Practices of health lifestyles

The young men’s differing dispositions to act and emotional states guided their practices. In our data we found practices of healthy lifestyles which enhanced the conditions for power and the ability to act, and also practices which restricted power and the ability to act.

#### Practices which enhance power

Practices and activities which enhance the conditions for power and the ability to act were examples of healthy lifestyles such as taking care of relations, devoting oneself to outdoor activities, planning for the future, changing destructive habits, and making oneself available for others’.

The most common practice was taking care of relations such as friends and family members. Having a girlfriend and spending time together with friends were described as important and pleasant activities. There were also narrations about helping family members or neighbors with various practical matters, such as tinkering with cars and other engines. These activities seemed to contribute to their picture of preferred masculinity. One man said:

Yes, I went to Paul and helped him with all kinds of things. Repairing, screwing and greasing everything possible.

Devoting oneself to outdoor activities such as hunting moose and small game in the autumn, driving scooters in the winter, or taking the dog out for a walk were practices that was used as alternatives to paid work when unemployed. Several of the young men seemed to find great pleasure in outdoor activities. Positive feelings like pleasure in spare time activities may spill over to enhanced activities in the labor market and thus to improved chances of finding employment.

Planning for the future can also be seen as a practice that enhances power. This was evident in such examples as saving money for a gun, a scooter, a musical instrument, a home of one’s own, or planning for one’s own business. Several of the young men had dreams and hopes for the future despite their problematic living conditions. One of them wanted to become an entrepreneur:

He and I have thought about opening a company of our own. I do not know how to arrange it—we will get someone to talk to the banks and so on for us. Than we have planned to buy [transport mopeds] while I work here we have been around and checked at various companies in town.

Changing destructive habits included cutting down on alcohol consumption and smoking, and living a quieter life. These healthy changes were often related to initiation of more stable relations. One young man expressed his healthier habits when moving together with his girlfriend in the following way; ‘And when you got a girlfriend then you have to calm down a little’. Other practices which gave meaning for the unemployed men were making one-self available to others, and assisting somebody with various tasks, e.g. shoveling snow in the winter and cutting lawns in the summer.

#### Practices which restricted power

In health practices which restricted power and ability to act there were various examples of unhealthy lifestyles such as having bad health habits and risk taking. Examples of bad health habits were smoking, poor food habits (e.g. irregular meals, too much saturated fat and simple carbs) and increased alcohol consumption.

R: How much did you drink?

*P*: *Every fortnight and sometimes I could drink three days per week … but then I drank nothing during a month*.

In response to a question of whether he drank more when he became unemployed, the man cited above answered: ‘No, I can hardly go out [due to lack of money]’. Sometimes, unemployment resulted in criminality: ‘And earlier when I was unemployed I was up to a lot of mischief… I mean, the police have caught me several times.’

Risk-taking and fearlessness were other characteristics in their masculinity construction. The intense search for a job combined with their focus on physically hard work often meant that safety precautions were set aside to the extent that some of them had been subject to work place accidents and complications resulting from accidents, violence and maltreatment.

No, I have head injuries and he knocked my head into those walls … he banged it, he hit my head first against the wall and then through the window…then I managed to flee out on the street but then he got hold of me again, he sat down on me and started to bang my head into the icy street…I have very bad memory after that

R (to another participant): Can you describe the sequel after the work accident?

P:… they say [about his pain]…I will always have it…the pain comes and goes. Clearly it is worse when I work.

Many of the practices, e.g. working with the body (such as working with vehicles, and hunting) and risk-taking behavior, can be attributed to the characteristic of the dominating masculinity among young working class men.

### Health experiences

The young men seemed to take their health for granted. Most of them declared that they were in very good health; ‘I feel like a prince’ was a common answer to the question ‘how do you feel?’ Other expressions related to health experiences can be referred to as ‘positive thoughts about and hope for the future’ and ‘making the best out of it’. Positive thoughts about the future and hope can be seen as a resource for a healthy lifestyle. One young man said ‘you want to try something new, that is what you need to do, that is when you look ahead. I believe it will be interesting [about getting a job]’. They seemed to experience meaning and hope, despite their sometimes problematic life situation.

‘Making the best out of it’ included, for example, statements about changes in lifestyle to a more healthy life. On the question ‘do you think you began to live an unhealthier and healthier life?’ one man answered:

Healthier, yes… I quit drinking or cut it down somewhat … when I became unemployed I calmed down a little. It was good [to be unemployed] because first it had been school which was somewhat stressful. Then you could calm down.

The most salient statement for unhealthy experiences described in the interviews was general ill-being, but also, headaches, sleeping difficulties, depression, allergies, and heart problems. There were narrations about general ill-being as being apathetic, lethargic and listless. ‘You get sick of unemployment, no question about it. In all ways: financially, physically and mentally’.

An example of a description of headache was: ‘Yes, in the morning … like a string like this [shows with his hand] but later in the day it is the whole forehead’. Another young man spoke of a headache as something that ‘it is pounding on both sides’ and that the headache never disappears. There were also narrations about difficulties sleeping. These sleeping difficulties were often related to going to bed late in the night which resulted in a vicious circle with long sleep-in and late night habits.

Descriptions of depression were often related to lack of money due to unemployment: ‘No, I had no money and was depressed and everything’. Problems related to diseases such as allergies were described: ‘Yes, I mean I have not had asthma before but I have had allergies all my life’. And also heart problems that meant a lot of visits to hospitals and health care centers:

Yes, I have started to get all possible problems with my heart again and I have started to seek care at the health care center as well as at the hospital.

Expressions related to health were not very prominent in the young men’s narrations, which is in line with the prevailing idea of masculinity among young men in the participants’ social context.

In relation to agency in structure models, our empirical data showed that despite that the young med shared the context of the macrostructure with unemployment and a disfavored social position, variations in their agency and construction of masculinities were apparent. The variations could mainly be referred to their different access to social support, described in life chances, which in turn influenced their life choices and dispositions to act.

## Discussion

Based on our theoretical point of departure from the ‘agency within structure model’, we find Cockerham’s Health Lifestyle Theory useful for interpreting our empirical findings. We agree with Cockerham [13] about the importance of restoring structure in qualitative research in order to increase our understanding of the importance of life circumstances and other social structures for enhancing or restricting power. Also, we found it valuable to introduce disposition to act (habitus) as a theoretical tool to capture the will and intentions of participants in health research. As a result of our empirical findings we believe that his model could be developed in the following ways (see Fig 1).

First, we visualize the macrostructure (class circumstances, trade and living conditions) as surrounding the whole model in an outer circle. In the same way, ‘socialization and experiences’ (including masculinities) can be visualized as an inner circle surrounding the inner components of the model. In this way we also hope to visualize that disposition to act is not only determined by the structures but is in dynamic interplay with them. This can be compared with for instance Sewell [4], who argues that *structure* ‘is dynamic, not static’ as an outcome of social interaction ([4], p.25)—and that *agency* arises from the (individual or collective) actor’s control of and mobilization of available resources ([4], p.20–21).

Second, while Cockerham mainly deals with lifestyle and health-related practices we also included health experiences (like illness as well as positive health aspects) as a component on the same level as ‘practices’. Other empirical testing may find different ways of dealing with health experiences, for example by integrating them into *all* parts of the model. In addition, we have found that disposition to act also includes important emotional and existential aspects, which would be of interest to further explore in future studies. We wonder why the Cockerham model has two separate boxes related to ‘healthy lifestyle’ (one called practices, another called health lifestyles). We have combined them into one component which we call ‘practices of health lifestyle’.

Third, and based on our inductive analysis, we have added power dimensions by analyzing each box in relation to what enhances or restricts power and ability to act. Drawing on Laverack [5–6], we regard the power dimensions as important, not only as an overall structure (power *over*) but also in relation to various other directions of power such as power from *within* (i.e. power on an individual level also known as sense of self-knowledge or inner strength), power *with* (used in for example relations between caregivers/practitioners and clients/others as a way of overcoming problems with ‘power over’) and powerlessness (when a habits of an individual cannot determine the outcome) [6]. Here we find disposition to act, reflecting perceptions and cognitive maps, to be central and to represent the young unemployed men’s incorporated habitus. In our results disposition to act was interpreted as either enhancing or restricting their power to act. In future studies these aspects are crucial to further address and develop.

Fourth, in recent work, Cockerham emphasizes the importance of structure for the thoughts, decisions, and actions (i.e. the agency) of individuals. According to him, structures do not predetermine but rather strongly influences agency but agency rarely exists without structure ([23], p.150). Our results differ from such a top-down approach as we cannot use findings from our qualitative analyses to draw firm conclusions about causal directions. Overall, the Cockerham model seems to be mainly developed for analyses of causal relations. We have empirically explored the content of each component of his model, while our analyses did not allow us to analyze pathways between the various components of the model. Such causal analyses can of course be made in future research. Our model can therefore be seen as a first step in the empirical testing of the Cockerham model. What we can say is that the possible pathways start with structural factors on societal level which provide the social context for socialization. Both of these provide the context for life chances and available resources like social support as well as to life choices like job searching activities which can lead to a cognitive map (for example being prepared for work) which in turn can be related to practices and health experiences.

Fifth, in contrast to Cockerham we have integrated a gender theoretical perspective in our paper by viewing the social constructions of masculinities as crucial parts of the socialization processes of young men. Our findings among the early unemployed young men can be interpreted as constructions of various young masculinities such as the hard-working, the risk-taking, the caring and connected, and the protesting and disconnected.

The young men who constructed the *hard-working masculinity* strived to fulfill the masculine ideal of working-class men in the region by accepting all available jobs, despite hazardous work environments in jobs that were available for uneducated young men on the labor market. Waged work was a prerequisite for their habitus, choices and agency. As a result, some were exposed to severe workplace accidents. As a comparison, social constructions of masculinity are also today commonly associated to risk-taking and being a real man [16]. Courtenay [24] argues that a risk-taking behavior is used in the daily ‘social structuring of gender and power’; and that the social practices which may undermine men’s health are used to negotiate social power and status. On the other hand, and in our analysis, constructions of social identity and value through work for others’ wellbeing and security were also interpreted as tied to experiences of being useful and *connected* to the community. This was a more health-promoting masculine position of caring and protection through hard work, in line with the collectively oriented welfare state ideas about responsibility for those unable to take care of themselves.

In contrast, the *disconnected masculinity* was related to disposition of pessimism and meaninglessness, as part of the young men’s scarce beliefs in having a future due to their marginalized position on the labor market. This masculine position could also be expressed in criminality or drugs use. In conjunction to this disconnected masculinity, a masculine position was revealed in relation to risk-taking and ‘anti-Joe-man’. The young men constructing the more independent or protesting masculinity were often ‘anti-school’ or ‘anti-Joe’ and strived to live a less traditional life. They opposed becoming a respectable family father and breadwinner by being independent, irresponsible and ‘anti-respectable’; not wanting to be part of societal norms and community. In comparison, Willis’ [25] studies of working class men in UK during the 1970’s describe masculine cultures that were violent, misogynist and anti-school. However, our results do not reveal a misogynist culture, but are more in line with Connell’s formulation of a *protesting masculinity* ([1], p.160), a stressed version of hegemonic masculinity explained as young unemployed men’s response to powerlessness. Connell ([1], p.155–156) views constructions of masculinities as ‘collective practice’ in their milieu of ‘historically constructed collective circumstances of life, in which effects of structure can be decoded, to which personal practice is addressed, but which is reducible to neither’. The term ‘milieu’ then, as Connell puts it, ‘goes beyond the categories of structure and action’ ([1], p.155). In the context of health promotion, Courtenay puts forward a need for protesting against the hegemonic masculinity including men’s dominant position in relation to women in order for men to decrease their risk-taking and improve their health behaviors [25]. Hence, gender-transformative public health interventions can have positive effect by challenging constraining masculine norms of e.g. ‘real men’ [26].

Frosh et al [27] suggest that changes in normative gender relations or in employment—which was the case in our study—mean that young men have to develop new forms of flexible, gendered, masculine identities, and that these are also expressed through social class position. How men construct their masculine identities therefore depend on time and place. Masculinities are from this viewpoint subject for change.

### Methodological considerations

In a qualitative study using content analysis, the trustworthiness and transferability of the results have to be considered. Qualitative studies are about understanding and conveying the sense people make of their life experiences. Our data were based on interviews with young unemployed men who were interviewed several times between age 16 and 22, a period of transition from youth to young adult a transition. The age span may be questioned as experiences at age 16 are likely to differ from those at age 22, but there are great individual variations in this process. The interviewees were asked to narrate their experiences in relation to being unemployed and their experiences of health. Although the interviewees were quite silent in their health experiences there were lots of reflections about life circumstances such as being unemployed as well as about the future. Earlier research in the field has also found that young men tend to be silent about their experiences of health and illness [28].

The first phase of our analysis was focused on unemployment. However, this uncovered the underlying meaning of powerlessness and also power, so the analysis proceeded to organize the findings in relation to powerlessness and power. This shift in approach can be seen as being undetermined. However, it could also be seen as strength in the meaning of being sensitive to what the text conveyed [29].

To establish trustworthiness, the inductive analysis was jointly carried out by two of the authors. The different steps in the analytical process were also discussed in the research group until consensus was achieved, thus strengthening the reliability of the interpretations.

Because this study included a small sample of ten men there is limited potential for generalizing the results to a larger group of young men. However, putting our finding into Cockerham’s model gave us the opportunity to theorize and contextualize our findings in light of prevailing living conditions, class and other collectivities. This contextualization increases the possibility of relating the findings to other groups of unemployed young men.

The interviews were performed in the early 1980ies and could be seen as a historical case study. However, those young men are adult today (in fact, they are 50 years old in 2015) and the period studied represent an important transition phase in young age between school and employment—as well as an political and societal break-point in the Swedish welfare state.

This study lays the foundation for understanding how a relatively supportive structure (with an active labor market policy in Sweden in the early 1980ies) could enhance both life chances, life choices and health while on the other hand, non-supportive structures could decrease power and ability to act and thus deteriorate health behavior during an important life phase. Constructions of masculinities at this time of life could have longstanding importance for adult health status as the foundation of health behaviors such as alcohol consumption is often laid in young age [30].

The context of Northern Sweden during the 1980s differs from the current situation in relation to classed and gendered norms, attitudes, and realities. As in many other parts of the Western world, the neoliberal politics have influenced the norms and attitudes of people of today in a more individualistic and market oriented way. The globalization has meant increased number of immigrants. In relation to structures, the unemployment rate has increased quite dramatically while the investment in labor market policy measures has not had the expansion needed to cover all unemployed. Gendered structures and relations, both in public and domestic arenas, have changed—and young masculinities and femininities are in flux. However, the fact that the world is changing does not mean that the situation 30 years ago is less interesting as it then laid foundation for agency and disposition to act as well as for construction of masculinities and other identities among those who are adult today.

### Conclusions

Qualitative research could contribute to develop the understanding of the agency within structure relationships. Cockerham’s Health Lifestyle Theory seems useful for analyzing what enhances or restricts power in an agency within structure framework. Disposition to act (habitus) seems to be a crucial theoretical tool to capture the will and intentions of individuals in relation to health. We have further developed the Cockerham model in the following ways:

Structure and socialization were visualized as surrounding the whole model. Analyses of what enhances or restricts power are important. In addition to practices of health lifestyle, we added experiences of health as outcome and emotional aspects in Disposition to act. We interpret our findings as construction of masculinities, in dynamic interplay with life chances and in relation to life choices, habitus and practices of health lifestyle.

Future studies need to pay attention to experiences of health among young people at the margin of the labor market in various milieus—and to analyze these in relation to gender constructions and within the frame-work of agency within structure.
